# Hardware Design and Implementation of a Wavelet De-Noising Procedure for Medical Signal Preprocessing

**DOI:** 10.3390/s151026396

**Published:** 2015-10-16

**Authors:** Szi-Wen Chen, Yuan-Ho Chen

**Affiliations:** 1 Department of Electronic Engineering, Chang Gung University, Taoyuan 333, Taiwan; E-Mail: chenyh@mail.cgu.edu.tw; 2 Healthy Aging Research Center (HARC), Chang Gung University, Taoyuan 333, Taiwan

**Keywords:** field programmable gate array (FPGA), medical signal preprocessing, electrocardiogram (ECG), signal integrity, wavelet de-noising

## Abstract

In this paper, a discrete wavelet transform (DWT) based de-noising with its applications into the noise reduction for medical signal preprocessing is introduced. This work focuses on the hardware realization of a real-time wavelet de-noising procedure. The proposed de-noising circuit mainly consists of three modules: a DWT, a thresholding, and an inverse DWT (IDWT) modular circuits. We also proposed a novel adaptive thresholding scheme and incorporated it into our wavelet de-noising procedure. Performance was then evaluated on both the architectural designs of the software and. In addition, the de-noising circuit was also implemented by downloading the Verilog codes to a field programmable gate array (FPGA) based platform so that its ability in noise reduction may be further validated in actual practice. Simulation experiment results produced by applying a set of simulated noise-contaminated electrocardiogram (ECG) signals into the de-noising circuit showed that the circuit could not only desirably meet the requirement of real-time processing, but also achieve satisfactory performance for noise reduction, while the sharp features of the ECG signals can be well preserved. The proposed de-noising circuit was further synthesized using the Synopsys Design Compiler with an Artisan Taiwan Semiconductor Manufacturing Company (TSMC, Hsinchu, Taiwan) 40 nm standard cell library. The integrated circuit (IC) synthesis simulation results showed that the proposed design can achieve a clock frequency of 200 MHz and the power consumption was only 17.4 mW, when operated at 200 MHz.

## 1. Introduction

Signal integrity always plays a crucial role for applications into medical signal sensing and processing. In general, determination of the use of an appropriate noise-reduction procedure in order to ensure the reliability for signal integrity is of paramount importance to the designs of the system-level medical products or devices. For example, a measured electrocardiogram (ECG) is mainly composed of actual cardiac activity and a variety of noise sources such as electromyogram (EMG), power-line interference and motion artifacts. In order to effectively perform the tasks of cardiac signal analyses, such as the QRS detection or arrhythmic event detection, simple and reliable signal preprocessing approaches for noise reduction are essentially demanded. In this regard, there are a number of traditional noise reduction methods, but most of them are usually operated in frequency domains with only limited frequency range. For instance, band-pass filtering has been known as one of the most common methods applied for this purpose. However, such a simple frequency-selective filtering operation may sometimes seriously destroy sharp features in ECG such as the QRS complexes [1]. On the other hand, in some existing cardiac signal processing such as the task of QRS detection for example, the correlation-based noise reduction method may be considered one of the best ways used to prevent the subsequent signal processing performance from being degraded by the undesired noise sources as described above [1]. However, since the technique involves intensive cross-correlation computation between a pre-selected QRS template and the raw ECG data, such a heavy computational burden could also undesirably restrict its use to quite a limited number of applications [2].

In fact, there actually exist a number of different types of other possible techniques for noise reduction. Among these methods, the wavelet based de-noising has been considered as one of the most effective techniques [3]. Since decades ago, wavelet-based methods have been widely used in biomedical signal processing [4,5]. In short, wavelet transforms represent the temporal characteristics of a signal by its spectral components in frequency domain. The theory of wavelet transforms asserts that signals to be analyzed can be decomposed into a variety of scales with different time and frequency resolutions using the so-called multi-resolution analysis algorithm [5,6]. Continued research investigations have addressed important issues across many applications spanning from data compression [7,8] to biomedical signal and image processing [9,10,11,12]. In addition to data compression, signal and image processing, it has been known that the wavelet transform-based technique can be also applied for the purpose of signal de-noising, as mentioned above. In this regard, the noise level can be effectively reduced while the sharp features or known noise characteristics of the signal can be well preserved [1,13,14]. For example, Du *et al*. have proposed a wavelet de-noising based method to well separate 1/f^γ^ noise from white noise in time domain with higher accuracy than classical frequency-domain based methods, even when the ratio of 1/f^γ^ noise to white noise or when the signal-to-noise ratio (SNR) is low [15].

In representing a signal that is contaminated by additive unstructured noise using discrete wavelet transform (DWT), we may hypothesize that most larger wavelet coefficients may generally result from the signal portion, while the small-valued wavelet coefficients should be attributed to the noise portion (which could possibly contaminate all wavelet coefficients). Thus, reconstructing the signal from the thresholded wavelet coefficients would provide a de-noised version of the original signal. Note that here the signal reconstruction is actually done by computing the inverse DWT (IDWT) [13,14]. Therefore, the fact as indicated above leads to the idea of de-noising a noisy signal in its wavelet domain. In addition, the DWT based de-noising method has been also widely applied in biomedical signal analysis [16,17,18]. Ahsan *et al*. have presented a fast and reliable prototyping of DWT as a VHDL (hardware description language) model for de-noising EMG signals [16]. Although their results indicated that the performance of the model was satisfactory, the computational cost in time and memory complexity could be greatly enhanced if higher precision is demanded. As for ECG applications, there was another work in literature demonstrating that the DWT was implemented using MATLAB to reduce the instrumentation and biological noise so the ECG parameters can be accurately estimated [17]. However, in that work, the de-noising scheme was only realized in software manner. Moreover, some researchers proposed to use the dual tree complex wavelet transform (DTCWT) to de-noise ECG signals [18]. Although they claimed that the DTCWT showed better performance for all types of noise than did the DWT, DTCWT actually requires high computational complexity and high redundancy in the output, which may not be desirable. In this paper, a novel, simple, and reliable wavelet-based de-noising algorithm and its hardware realization for real-time medical signal preprocessing and analysis is presented. This study mainly evaluated the proposed DWT de-noising method and its circuit architecture in both software and hardware manners. It is also worth noting that in such a circuit, the real-time implementation of DWT computation requires substantially smaller memory storage, as compared to that required by the direct computation of DWT, thus permitting a time- and memory-efficient architecture. Simulation experiment results obtained from both the software and hardware simulations indicated that the proposed wavelet de-noising scheme could robustly and effectively reduce the noise level in a real-time manner.

## 2. Methodology

### 2.1. Overall Structure

**Figure 1:**
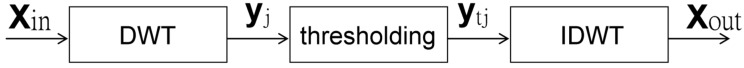
A schematic block diagram of the wavelet de-noising process.

Figure 1 shows a schematic block diagram of the overall wavelet de-noising process. According to Figure 1, the procedure of the wavelet de-noising scheme is briefly described as follows. First, the three-level, four-tap DWT of a noise-contaminated medical signal is computed. Next, a so-called thresholding process is applied to the DWT by throwing away the wavelet coefficients (*i.e*., resetting them to zero) whose absolute magnitudes are less than some preset threshold [13,14]. The rationale behind the thresholding process is based on an assumption that the noise generally results in small-valued wavelet coefficients while the large coefficients should be mostly due to the actual signal. Then, the de-noised signal can be synthesized simply by computing the IDWT on the thresholded wavelet coefficients and thus the SNR of the de-noised signal is finally enhanced. In short, the wavelet de-noising circuit to be designed here in this study mainly consists of three modules: a DWT, a thresholding, and an IDWT, which are respectively presented in the subsequent sections.

### 2.2. First Stage: DWT

**Figure 2:**
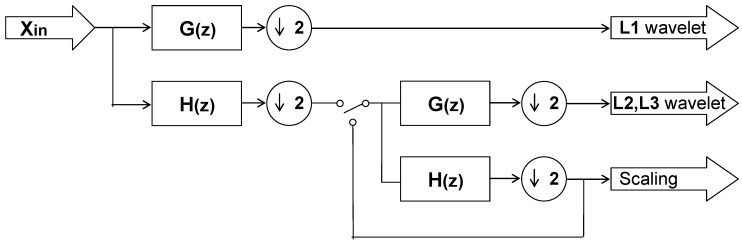
A three level 1D DWT structure as developed in [21].

In this section, a real-time DWT structure is briefly described. First, note that computing DWT in a real-time fashion with low memory complexity is desirable in many aspects of medical signal processing applications. On the other hand, being implemented by convolution, the DWT computation requires both a large number of arithmetic operations (*i.e*., multiplication and addition) and a large storage in memory, which are not desirable for high-speed or low-power signal or image processing applications. In fact, direct computation of an N-point DWT, referred to as the Pyramid Algorithm (PA) [19,20], requires *O*(*N*) storage in memory and this obviously costs too much. Meanwhile, to achieve real-time wavelet de-noising, it is neither practical nor possible to directly compute the DWT for the entire signal that is supposed to be quasi-infinite in length. Fortunately, there were a number of efficient scalable VLSI (Very-large-scale integration architectures) for real-time computation of DWT developed previously [21,22,23,24,25]. Among all these efficient VLSI architectures, a structure referred to as the lifting-based scheme demands much fewer computations than do the conventional ones and thus has been proposed for the DWT implementation for the past several years [24,25]. However, since the lifting scheme can be only applied for constructing biorthogonal wavelets, it may not be well suited to our applications. Therefore, in this study an existing structure of one-dimensional (1D) DWT decomposition developed by Yu *et al*. [21] was thus incorporated into the de-noising circuit, as shown in Figure 2. Unlike the lifting based scheme, the DWT architecture we adopted here can be applied for constructing any wavelet filters.

Note that since here the Daubechies 4 filters were adopted, *H*(*z*) and *G*(*z*) denote a 4-tap lowpass and highpass filters, respectively, where

(1) $$\begin{array}{l} G(z)=g_{0}z+g_{1}z^{-1}+g_{2}z^{-2}+g_{3}z^{-3}\\ H(z)=h_{0}z+h_{1}z^{-1}+h_{2}z^{-2}+h_{3}z^{-3} \end{array}$$

**Figure 3:**
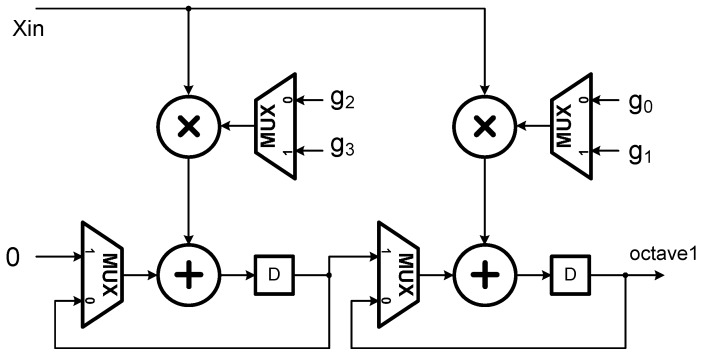
The first stage architecture of the 1D DWT as developed in [21].

**Figure 4:**
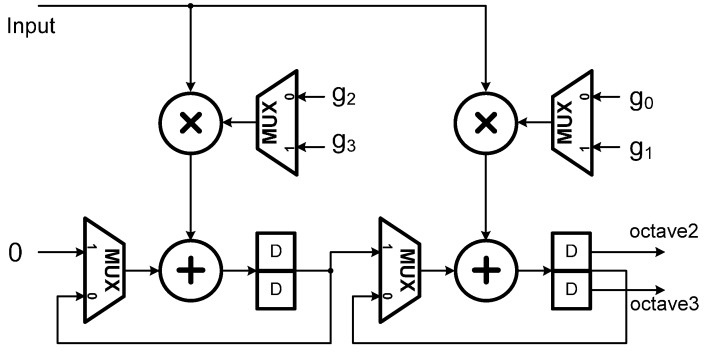
The second stage architecture of the 1D DWT as developed in [21].

According to [21], computations of all the wavelet coefficients after the second level can be folded into the second stage filter bank in Figure 2 by interleaving the computations of the second level with those of the remaining levels (note that there are three dyadic levels in this study). Such an operation scheduling approach results in the architectures of the first and second stages for realizing the decimation filter process *G*(*z*) as indicated in Figure 3 and Figure 4, respectively. Similarly, *H*(*z*) filtering operations can be realized in the same way.

From the architectures as shown in Figure 3 and Figure 4, one may see that the number of Multiplications and Accumulations (MAC’s) is reduced by exactly 50%, in comparison to the conventional cases [21]. Consequently, for an M-tap filter, the total number of MAC’s in the architecture adopted in this study is 2M (*i.e.*, 2 modules/stage × M/2 MAC’s/module × 2 stages) which is the same as most of the existing folded architectures; however, the adopted folded architecture can provide an ideally high throughput rate. Moreover, for a J-level DWT the architecture requires only JM registers, even much less than the conventional ones (note that in our case, M = 4, J = 3).

### 2.3. Middle Stage: Thresholding

As stated previously, given a raw measured signal, in order to effectively reduce the noise level and simultaneously preserve the sharp features of the signal, a deliberately designed thresholding stage needs to be incorporated into the de-noising circuit. In this study, a novel adaptive thresholding scheme was devised. Such a thresholding process is applied to the wavelet coefficients generated from the DWT module for reducing the noise level while preserving the sharp features of the signal. It should be noted that there was an adaptive threshold assigned for each DWT level (or dyadic level) and used to perform the thresholding process on the wavelet coefficients generated at that level. Here, the adaptive threshold applied for the *j*-th dyadic level is defined as

(2) $$th_{j}(i)=\frac{1}{32}\sum_{k=32(i-1)}^{32i-1}\left|y_{1}(k)\right|\times 2^{j}$$

where *y*_1_(*k*) represents the wavelet coefficients at the first dyadic level; *th_j_*(*i*) represents the *i-*th threshold value used for performing the thresholding process on the wavelet coefficients at the *j-*th level. Obviously, the thresholds used for different levels were all related one another because according to Equation (2) they were all determined using the wavelet coefficients at the 1st level. In order to explain the calculation of the thresholds more clearly, first we consider the threshold for the 1st level (*i.e*., let j = 1).

(3) $$th_{1}(i)=\frac{1}{32}\sum_{k=32(i-1)}^{32i-1}\left|y_{1}(k)\right|\times 2$$

Or alternatively, we have $th_{1}(1)=\frac{1}{32}\sum_{k=0}^{31}\left|y_{1}(k)\right|\times 2,$ $th_{1}(2)=\frac{1}{32}\sum_{k=32}^{63}\left|y_{1}(k)\right|\times 2,$ $th_{1}(3)=\frac{1}{32}\sum_{k=64}^{95}\left|y_{1}(k)\right|\times 2,$ obviously, we may see that the adaptive threshold *th*_1_(*i*) applied for the 1st level DWT coefficients was simply determined and updated by the running estimate of twice as much as the average of the absolute 1^st^-level wavelet coefficients over a 32-point non-overlapping window. Here, 32 is an empirical choice of the moving window length. Furthermore, according to Equation (2) we then have

(4) $$th_{2}(i)=th_{1}(i)\times 2$$

And

(5) $$th_{3}(i)=th_{2}(i)\times 2=th_{1}(i)\times 4$$

That is, the threshold would be elevated by a factor of 2 as the dyadic level is increased by 1. Therefore, the threshold *th_j_*(*i*) is updated every 32 first-level wavelet coefficients. Also note that since the scaling factor (*i.e*., 2*^j^*/32) in Equation (2) can be represented as a power of 2, the computation in Equation (2) is thus accomplished by performing a number of additions first and then a simple shift operation at the end. Moreover, the thresholding scheme can be formulated as

(6) $$\begin{array}{l} y_{tj}(k)=y_{j}(k),\text{ if }\left|y_{j}(k)\right|\geq th_{j}(i),\\ y_{tj}(k)=0,\text{ if }\left|y_{j}(k)\right|<th_{j}(i) \end{array}$$

where *y_tj_*(*k*) represents the new value of the *k*-th wavelet coefficient at the *j*-th level obtained after thresholding, where *k* = *B_j_*(*i* − 1) ~ *B_j_i*−1 (*B_j_* = 32/2^(*j*−1)^). Note further according to Equations (2) and (6), one may see that the threshold is applied and updated in a block-wise manner. Therefore, in order to synchronously perform the thresholding process on the wavelet coefficients throughout all levels, a threshold *th*_2_(*i*) is applied for the *i*-th block consisting of 16 consecutive wavelet coefficients at the second level (*B*_2_ = 16); similarly, *th*_3_(*i*) is applied for the *i*-th block consisting of 8 consecutive wavelet coefficients at the third level (*B*_3_ = 8).

**Figure 5:**
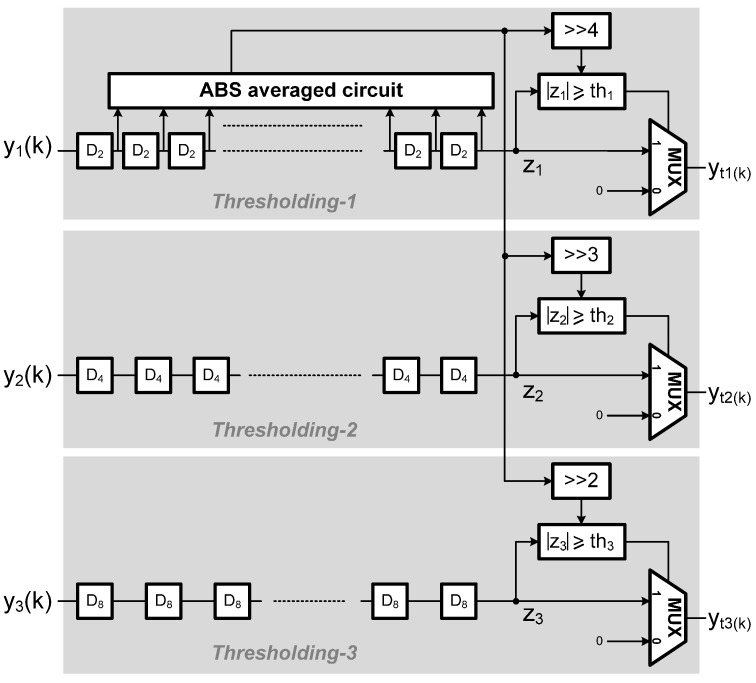
The architecture of the proposed thresholding circuit.

Figure 5 shows the architecture of the proposed thresholding circuit. Three computation paths respectively filter three level data (*i.e*., wavelet coefficients) obtained from the DWT output based on the corresponding thresholds in a nonlinear manner, as indicated in Equation (6). The threshold calculation consists of two steps described as follows. First, the average of the absolute values of 32 consecutive wavelet coefficients at the first level is computed using an ABS averaged circuit. The thresholds used for all the three computation paths, denoted as *th*_1_(*i*), *th*_2_(*i*), and *th*_3_(*i*), can be then obtained by right shifting 4-bit, 3-bit, and 2-bit, respectively. In addition, the shift registers D_2_, D_4_, and D_8_ permute the data sequences derived from the DWT and feed them into the inverse DWT after the thresholding process. Due to the down sampling of the DWT and up-sampling of IDWT operations, their update frequencies are 1/2, 1/4, and 1/8 fold, respectively, to the system clock, and thus, the proposed adaptive thresholding scheme, as indicated in Equations (2) and (6), can be successfully executed by the proposed circuit. Note further that due to the algorithmic regularity, the proposed thresholding architecture can be easily scaled up with the block size and the number of dyadic levels.

### 2.4. Final Stage: IDWT

**Figure 6:**
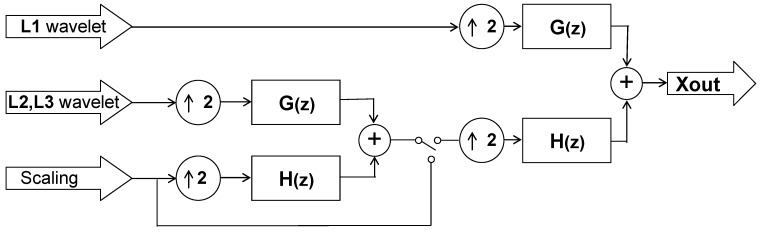
A three level 1D IDWT structure.

**Figure 7:**
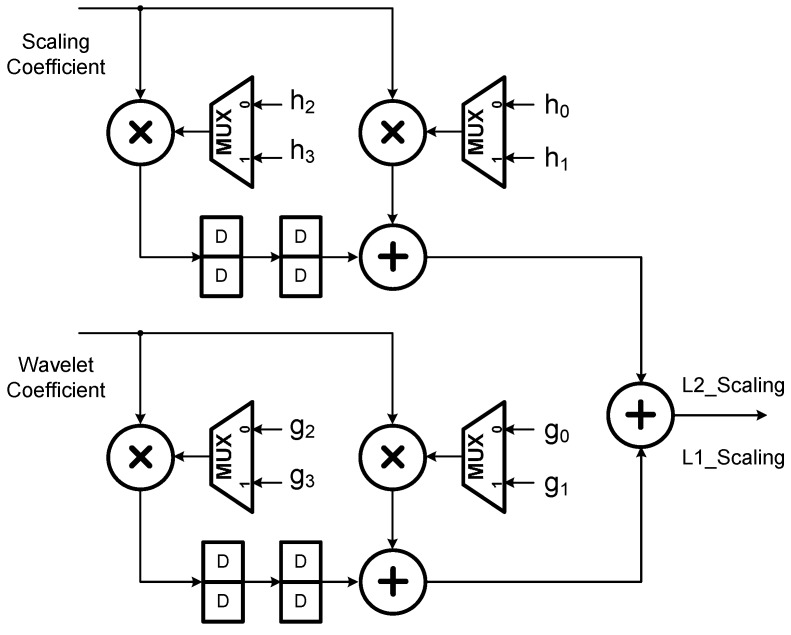
The first stage architecture of the 1D IDWT. Note that the output of the circuit is switched between the scaling coefficient input of this circuit itself and the coarse coefficient input of the next stage circuit (*i.e*., the second stage circuit, as depicted in Figure 8).

**Figure 8:**
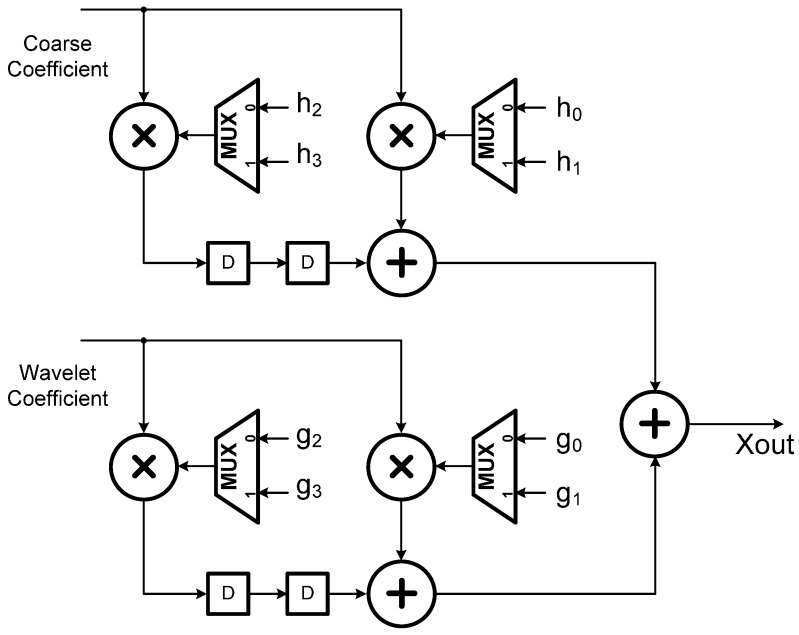
The second stage architecture of 1D IDWT.

**Figure 9:**
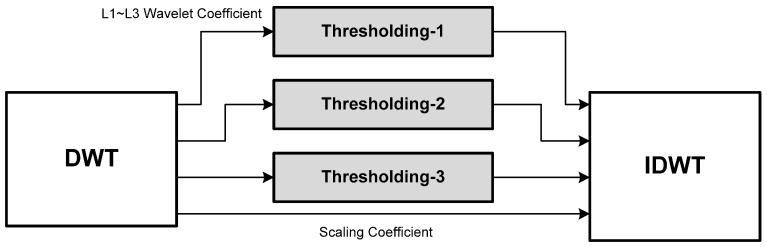
A schematic block diagram of the three-level wavelet based de-noising structure.

At the final stage, the de-noised signal is generated by computing the inverse DWT (IDWT) on the thresholded wavelet coefficients. Figure 6 gives an illustrative structure of the three-level 1D IDWT synthesis. In fact, the computation of all the scaling (or coarse) coefficients over all levels can be folded into the first stage filter bank as shown in Figure 6. This folded structure is illustrated in Figure 7. Moreover, given the first level scaling and thresholded wavelet coefficients, the de-noised signal is then synthesized at the second stage filter bank, as illustrated in Figure 8. With a switch-based connection of the architectures of Figure 7 and Figure 8, the IDWT module derived for this research would work perfectly in accordance with the preceding DWT and thresholding modules to produce the de-noised signal in a real-time manner. Figure 9 depicts a schematic block diagram illustrating how the DWT, the thresholding, and the IDWT modules connect to perform the de-noising function.

In addition, it should be also noted that there are a number of thresholded wavelet coefficients obtained after applying the thresholding process throughout all the levels waiting to be input into the IDWT module, and thus, one may expect that the number of registers required for the implementation of the wavelet de-noising algorithm as shown in Figure 9 would be greatly increased. Therefore, the focus of the next step design is on minimizing the number of registers used in the circuit so the chip size due to the registers remains optimally small. For this purpose, here a lifetime analysis used to systematically evaluate the minimum number of registers was further adopted. That is, after the minimum number of registers required for the implementation of the circuit was determined, a technique, referred to as the forward-backward register allocation, was then used for allocating the intermediate thresholded wavelet data to these registers [26,27].

## 3. Simulation Experiment Results of Performance Evaluation and FPGA Implementation

To evaluate the performance of the proposed de-noising algorithm as well as to demonstrate its noise reduction capability under a field programmable gate array (FPGA) design, simulation experiments were conducted in both software and hardware manners. It should also be noted that since a practically measured ECG signal is mainly composed of actual cardiac activity and noise due to various sources, such as electromyogram (EMG), power-line interference and motion artifacts. In this performance evaluation task, we consider the de-noising performance upon the white Gaussian noise first, and then upon the 60 Hz power interference when combined with white Gaussian noise, which can be thought of as being a more realistically encountered type of noise.

### 3.1. Simulation Results of White Gaussian Noise Reduction

**Figure 10:**
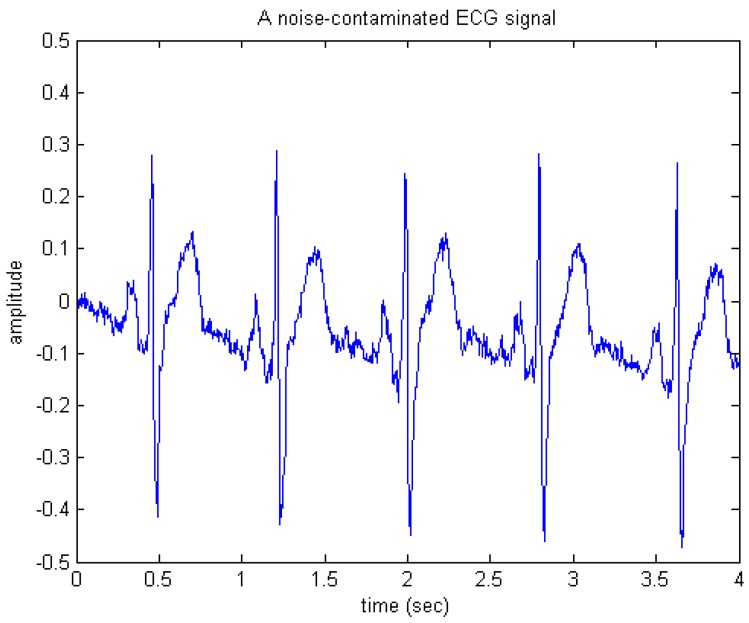
A simulated noise-contaminated electrocardiogram (ECG) signal.

Here, given a noiseless ECG signal, a simulated noise-contaminated ECG signal was then composed by adding a zero-mean white Gaussian noise signal to the noiseless ECG. The sampling rate of the ECG signal was 250 Hz. Figure 10 shows an example of the simulated Gaussian noise corrupted ECG signal used in this performance validation task.

**Figure 11:**
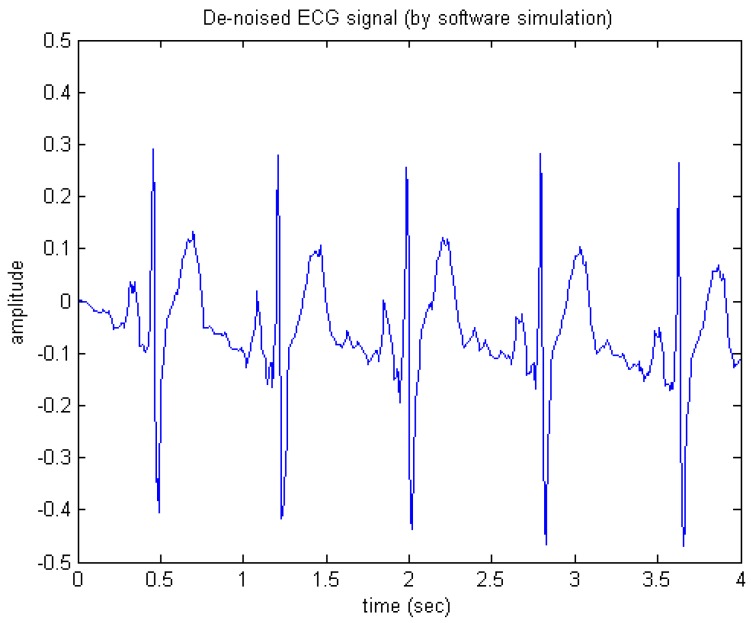
De-noised ECG signal (by software simulation).

First, in software/floating-point simulation the proposed de-noising algorithm was realized using MATLAB codes and then tested on the raw ECG signal as shown in Figure 10. Figure 11 shows the appearance of the output generated simply by executing the MATLAB codes. Apparently, the de-noised signal as shown in Figure 11 looks much cleaner and thus, most of the important features or components of ECG may be clearly observed, indicating that the wavelet de-noising could faithfully and robustly recover the desired ECG signal from the noise-contaminated situation.

**Figure 12:**
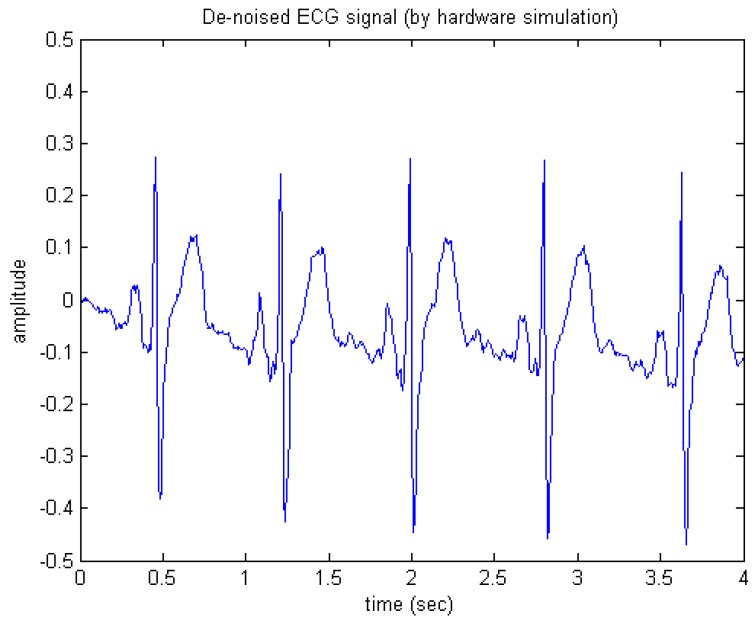
De-noised ECG signal (by hardware simulation).

Next, as for the hardware/fixed-point simulation, we realized the de-noising circuit using the Verilog Hardware Description Language (Verilog/HDL) and then simulated the circuit. Figure 12 shows the hardware simulation results. The fixed-point word length of the circuit was set to 11 bits plus one sign bit. In fact, the correlation coefficient between both the software and hardware de-noised ECG signals is over 0.99.

We also implemented the circuit on a commercial PC-based high-speed field programmable gate array (FPGA) platform, called the SMIMS VeriEnterprise (SMIMS Technology Corporation, Taipei, Taiwan) so that its actual processing capability in the form of hardware may be faithfully validated. The SMIMS VeriEnterprise is a PC-based high-speed FPGA platform. It provides a hardware-software co-design, co-simulation, and co-verification environment and is thus very suitable for a complete application specific integrated circuit (ASIC) logic emulation, prototyping verification and IP development. In such a hardware experimental platform, we imported the input signals from PC into our designed circuit on the FPGA of VeriEnterprise and exported the output signals to PC for observation. Consequently, the de-noised ECG signal generated by the FPGA module was exactly the same as that obtained from the hardware simulation results as depicted in Figure 12. It should be noted that the design and testing procedure, as described above, is also useful in designing either a stand-alone processor, or a coprocessor that may work in conjunction with medical embedded systems and/or systems on a chip.

### 3.2. Simulation Results of 60 Hz Power Interference Mixed with Gaussian Noise Reduction

**Figure 13:**
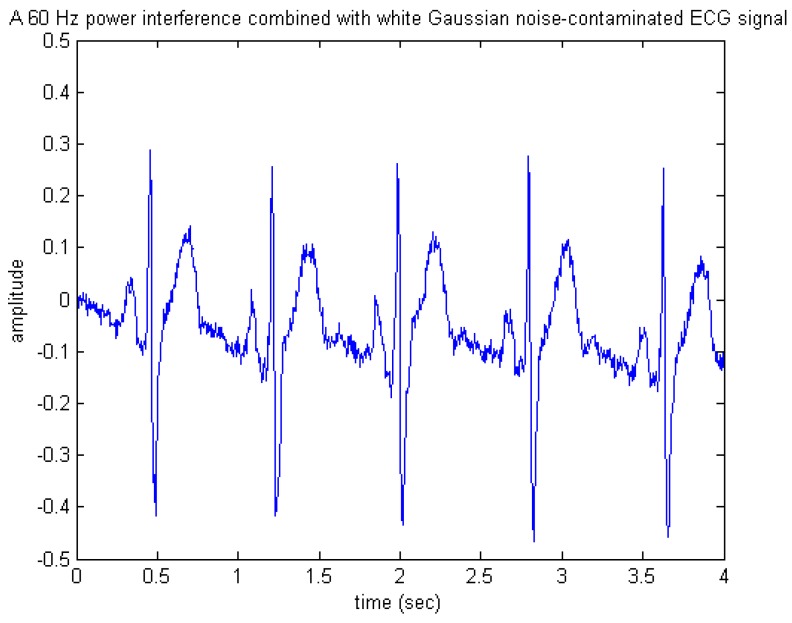
A simulated 60 Hz power interference mixed with white Gaussian noise-contaminated ECG signal.

Since, as stated previously, raw ECG usually presents different kinds of noise sources such as EMG, 60 Hz power-line interference, and motion artifacts, only white Gaussian noise used in the performance evaluation may limit the extent and scope of the investigation. Thus, it is necessary to include other noise types in the performance evaluation. Here, simulation results of a mixed 60 Hz power line interference and Gaussian noise reduction are presented. Similarly, given the same noiseless ECG signal, a simulated 60 Hz power interference mixed with white Gaussian noise-contaminated ECG signal was thus composed, as depicted in Figure 13.

**Figure 14:**
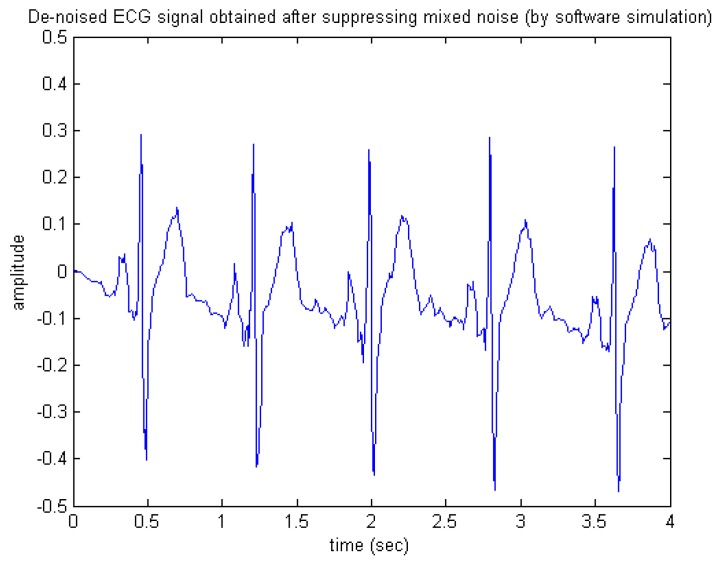
De-noised ECG signal obtained after suppressing mixed noise (by software simulation).

**Figure 15:**
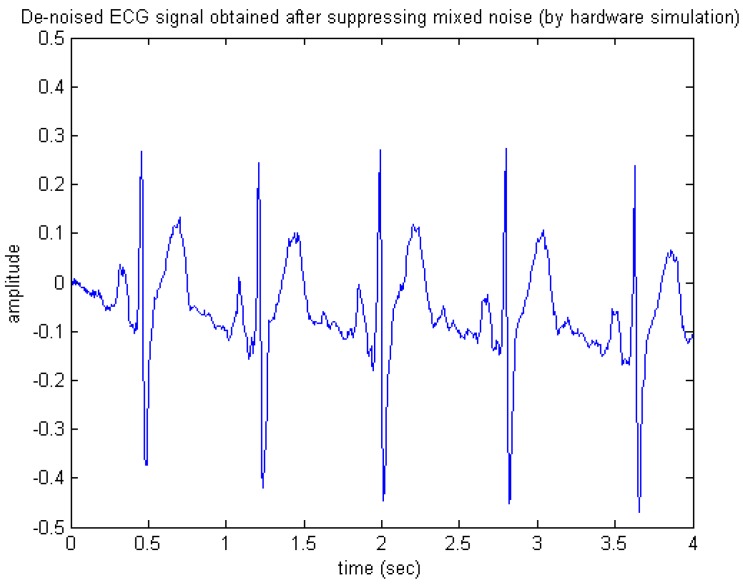
De-noised ECG signal obtained after suppressing mixed noise (by hardware simulation).

Figure 14 and Figure 15 present the appearances of the de-noised output signals produced by the software and hardware simulations, respectively. Similarly, observing either Figure 14 or Figure 15, one may see that most of the important features of ECG were preserved, thus indicating that the wavelet de-noising could still robustly recover the desired ECG signal from the noise-contaminated situation, even under the presence of the 60 Hz power-line interference.

### 3.3. Performance Evaluation and Discussion

In order to understand how the SNR affects the performance of the proposed DWT based de-noising architecture, we experimentally studied its de-noising performance on a set of simulated noisy ECG signals at a variety of SNR levels ranging from 0 to 12 dB. As described previously, we had taken a case where the simulated ECG measurement was a composite signal of a noiseless ECG and a zero-mean white Gaussian noise with variance σ^2^. That is, given a noiseless ECG, each simulated noise-contaminated ECG signal with a preset SNR value (ranging from 0–12 dB) can be then composed by adding a white Gaussian noise signal with the estimated variance *σ*^2^ (corresponding to the given SNR value) to the noiseless ECG. Both the de-noising algorithmic and its architectural performances were evaluated against all these simulated ECG measurements (with different SNR values). That is, we tested and examined the capability of the proposed DWT de-noising in its software and hardware realizations. For this purpose, a quantitative measure, referred to as the Percent Error Energy (PEE), was further used. It is defined as the percentage of the magnitude-squared difference between the noiseless ECG and the de-noised ECG, that is,

(7) $$\text{PEE }=\frac{{\left\|x-x_{0}\right\|}_{2}^{2}}{{\left\|x_{0}\right\|}_{2}^{2}}\times 100\%$$

where x and x_0_ represent the de-noised ECG and the noiseless ECG, respectively. In addition, a similar metric typically used for quantitatively evaluating the de-noising performance, called the percent root mean square difference (PRD) can be also used for the performance evaluation [28,29]. In fact, PRD can be simply estimated from PEE using

(8) $$\text{PRD }=\sqrt{\text{PEE}}\times 10$$

**Table 1:** SNR *versus* the PEE values obtained before applying wavelet de-noising, after applying software- and hardware-based wavelet de-noising, respectively, *i.e*., the numerical results as shown in Figure 16. Note that in order to make a performance comparison between the proposed method (and its circuit) and an existing de-noising method [28], we here also included the percent root mean square differences (PRDs) when SNR levels were at 6 dB and 10 dB, respectively.

SNR (dB)	PEE (%)		
	Before De-Noising	After De-Noising (SW)	After De-Noising (HW)
0	71.4768	20.2165	35.4926
1	55.3751	11.5416	26.8590
2	47.1088	14.7589	24.1877
3	38.1031	10.2151	20.6781
4	29.8481	6.8174	16.5251
5	24.3355	7.8158	13.1456
6	18.9428	6.4032 (PRD ≒ 25%)	10.2769 (PRD ≒ 32%)
7	14.6350	5.1588	8.2892
8	11.5079	4.8156	6.9530
9	9.4788	3.0588	5.9702
10	7.1918	2.3661 (PRD ≒ 15%)	4.9530 (PRD ≒ 22%)
11	5.5625	1.9624	4.0578
12	4.8982	1.5295	3.7200

**Figure 16:**
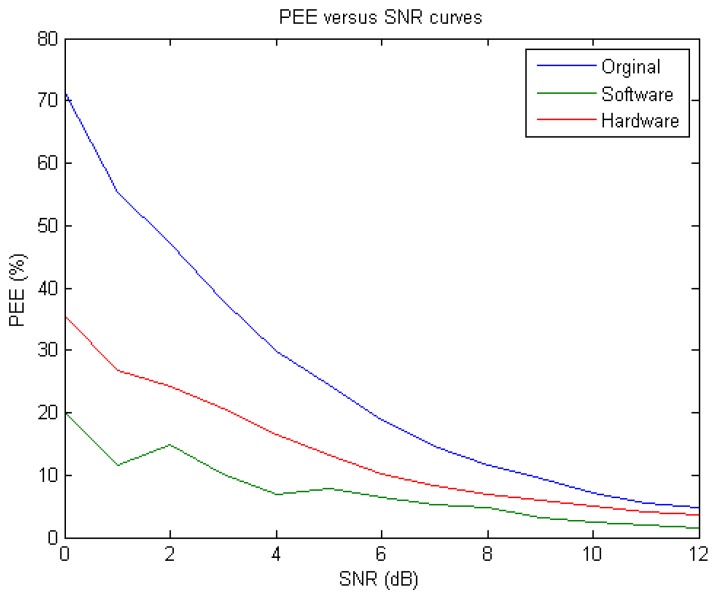
The Percent Error Energy (PEE)-*versus*-signal-to-noise ratio (SNR) curves derived from the original noise-corrupted ECGs (blue), the software-based de-noised ECGs (green), and the hardware-based de-noised ECGs (red), respectively.

In fact, at a particular SNR, a de-noising method is said to be better if both the PEE and PRD are smaller. Note that in order to compare the performances obtained before and after applying the proposed DWT based de-noising scheme, we also evaluated the PEE between each original noise-contaminated ECG signal and the noiseless ECG simply by letting x in Equation (7) be the original raw ECG. Figure 16 presents the performance comparison in terms of PEE-*versus*-SNR curve among all the cases corresponding to the original raw ECG, the software-based de-noised ECG, and the hardware-based de-noised ECG, respectively. Observing the results as shown in Figure 16, one may see that the as expected, the PEE values evaluated on the original noise-contaminated ECG signals were all greater than those evaluated on the de-noised signals, regardless of being derived in hardware or in software manner. Also, one may notice that the software realization substantially outperformed the hardware one. This is because the former leads to the floating-point representations while the latter is in fixed-point implementation. In our case, the fixed-point word length was set to 12 bits. In fact, in hardware realization the desire for fine quantization (*i.e.*, small quantization error) can be done simply by increasing the fixed-point word length [30]. In addition, all the numerical results of PEE obtained at different SNR values are also tabulated in Table 1. We further compared the performance of our de-noising algorithm and circuit, as shown in Table 1, with respect to an existing method, as proposed in a previous work [28], based on PRD. According to [28], they obtained about 48% of PRD at an input SNR level of 6 dB and about 40% of PRD at SNR of 10 dB, while our algorithm achieved only about 25% and 32% of PRDs in software and hardware realizations, respectively, at SNR of 6 dB and about 15% and 22% at SNR of 10 dB. Obviously, the proposed de-noising algorithm and its circuit both achieved much better de-noising performance than did the one as proposed by [28].

**Table 2:** SNR *versus* the PEE values obtained before applying wavelet de-noising, after applying software- and hardware-based wavelet de-noising, respectively, *i.e*., the numerical results as shown in Figure 17. Similarly, we here also included the PRDs when SNR levels were at 6 dB and 10 dB, respectively, for performance comparison with respect to [28].

SNR (dB)	PEE (%)		
	Before De-Noising	After De-Noising (SW)	After De-Noising (HW)
0	78.8927	22.9218	48.1399
1	62.8969	18.7075	38.8732
2	45.1561	11.3090	29.0159
3	34.6861	5.9853	22.6072
4	30.2524	6.6459	19.8421
5	22.4941	5.7056	14.7440
6	17.8927	3.9962 (PRD ≒ 20%)	11.6299 (PRD ≒ 34%)
7	15.4273	3.5649	10.4252
8	11.5474	3.4916	8.0020
9	8.8930	2.1120	6.6262
10	7.2446	2.1843 (PRD ≒ 15%)	5.5503 (PRD ≒ 24%)
11	6.0442	1.7293	4.7493
12	5.0159	1.0922	4.2236

**Figure 17:**
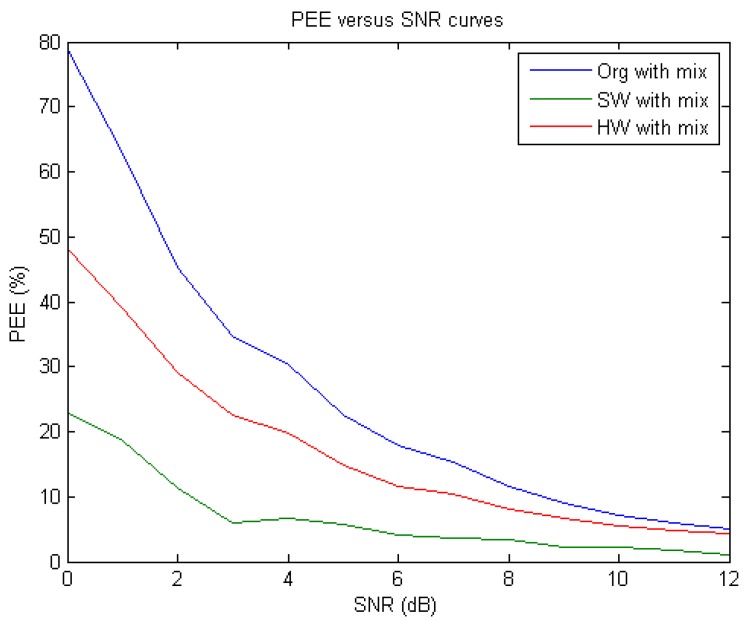
The PEE-*versus*-SNR curves derived from the original 60 Hz power interference and Gaussian noise-corrupted ECGs (blue), the software-based de-noised ECGs (green), and the hardware-based de-noised ECGs (red), respectively.

Furthermore, we also experimentally studied the de-noising performance on a set of simulated 60 Hz power interference and Gaussian noise-contaminated ECG signals at different SNR levels (ranging from 0 to 12 dB) and all the numerical results of PEE obtained at different SNR levels were depicted in Figure 17 and tabulated in Table 2. Similarly, we further compared the performances of our de-noising algorithm and circuit respectively, with respect to the existing method [28] based on PRD. Again, inspecting Table 2 we may see that our algorithm achieved only about 20% and 34% of PRDs in software and hardware realizations, respectively, at SNR of 6 dB and about 15% and 24% of PRDs at SNR of 10 dB, which were both much smaller than those obtained from the method as proposed by [28] at the same levels of SNR (Recall in [28], they achieved about 48% of PRD at SNR level of 6 dB and about 40% of PRD at SNR of 10 dB). As a result, the proposed de-noising algorithm and its circuit still showed much better de-noising performance than did the one as proposed by [28].

Finally, in order to further demonstrate the proposed design, the Synopsys Design Compiler was also applied with an Artisan TSMC 40 nm standard cell library to implement the entire de-nosing system. The performance characteristics of the integrated circuit (IC) synthesis simulation are as listed in Table 3. The results showed that the proposed design can achieve a clock frequency of 200 MHz with 25,082 μm^2^ in chip area, and the power consumption was only 17.4 mW, when operated at 200 MHz.

**Table 3:** Performance characteristics of the IC synthesis simulation for the proposed de-noising system design.

Performance Characteristics	
Technology	40 nm
Supply Voltage	1.2 V
Circuit Area	25,082 μm^2^
Frequency	200 MHz
Power Consumption	17.4 mW

## 4. Conclusions

In this paper, the implementation of a real-time DWT based signal de-noising algorithm is introduced. The aim of this study is to devise and realize a digital signal processing (DSP) architecture associated with the wavelet de-noising scheme, thus ensuring and enhancing the microelectronic reliability for signal integrity in the system-level medical devices or products of interest. The proposed wavelet based de-noising architecture is constructed by a cascade combination of three modules: a DWT, a thresholding, and an IDWT. Simulation experiment results obtained after performing both the software and hardware simulations on the task of ECG de-noising demonstrated the feasibility of the proposed DSP architecture. In addition, one may see that the circuit could not only meet the requirement of real-time processing, but also effectively reduce the noise level associated with the ECG signal while the sharp features or components of the medical signal can be faithfully preserved. Finally, we successfully implemented it on a commercial FPGA chip for prototyping and real-time applications. In fact, the proposed de-noising FPGA circuit may serve as a coprocessor on an embedded medical signal analysis system, or be integrated into a multi-functional medical system-on-chip (SoC) in near future.
